# From Laurie Todd

**Published:** 1857-11

**Authors:** Grant Thorburne

**Affiliations:** New Haven


### FROM LAURIE TODD'S NOTE-BOOK.

I WAS only thirty months old when my mother died. By the neglect of a servant, I lost the use of my limbs, and sat nearly a twelvemonth on a small bench, looking at the fire. One day a gipsy entered, and asked for something to eat. While she was taking some milk and bread, my small figure drew her attention. She inquired into my case, and advised my father to board me on a heather-hill near by, which abounded with small snails, the shells of which were half an inch long and beautifully striped. The snails came out of their holes every morning about sun-rise. I was sent out to gather a cup-full. They were washed, boiled in milk, fresh from the cow. This milk was given me to eat with my oat-meal porridge, and was very palatable. I was much amused at gathering the snails; the exercise imparted strength to my limbs, while the snail-soup nourished my feeble frame. Now, I walk without a staff.

When in my twelfth year, my father consulted Dr. Monro, of Edinburgh, whether to apprentice me to a tailor or a wrought-nail maker. The nail-maker stands upright while at work, with the whole body in gentle motion. The hammer was put into my hand in my twelfth year. From that day my health and strength began to improve; and now, when my sun is sinking in the west, my personal feelings are as comfortable as they were sixty years ago, seeing and hearing excepted.

Parents, nurses, and physicians err when they place a weak, lame, and rickety boy on a tailor's board, or a shoemaker's stool; better buy his coffin at once.

All the works of God are in perpetual motion. If men wish health and comfort, they must keep moving.

From 1794 to 1802, I made wrought-nails in New York, when the cut-nails appeared, and put me out of employment.

GRANT THORBURNE, SR.

NEW HAVEN, *Sept.* 30, 1857.

